# 1-Methyl-5-nitro­imidazolium chloride

**DOI:** 10.1107/S2414314622008781

**Published:** 2022-09-08

**Authors:** Sophia Bellia, Grace Anderson, Matthias Zeller, Arsalan Mirjafari, Patrick C. Hillesheim

**Affiliations:** a Ave Maria University, Department of Chemistry and Physics, 5050 Ave Maria Blvd, Ave Maria, Florida 34142, USA; b Department of Chemistry and Physics, Florida Gulf Coast University, 10501 FGCU Blvd. South, Fort Myers, Florida 33965, USA; c Purdue University, Department of Chemistry, 560 Oval Drive, West Lafayette, Indiana 47907, USA; d Department of Chemistry, State University of New York at Oswego, Oswego, New York 13126, USA; Sunway University, Malaysia

**Keywords:** crystal structure, imidazolium, π-hole, hydrogen bonding

## Abstract

The title salt, C_4_H_6_N_3_O_2_^+^·Cl^−^, exhibits multiple hydrogen-bonding inter­actions involving the nitro­imidazolium cation and the chloride anion. Strong hydrogen bonds between the amine hydrogen atom and the chloride anion link the ionic moieties. Of note, with respect to H⋯Cl inter­actions, the central aromatic hydrogen atom displays a shorter inter­action than the other aromatic hydrogen atom. Finally, inter­actions are observed between the nitro moiety and methyl H atoms. While no π–π stacking is observed, anion-π inter­actions are present. The crystal was refined as a two-component twin.

## Abstract 2

A combination of hydrogen-bonding and anion-π inter­actions help form the extended structure of the title salt.

## Graphical abstract

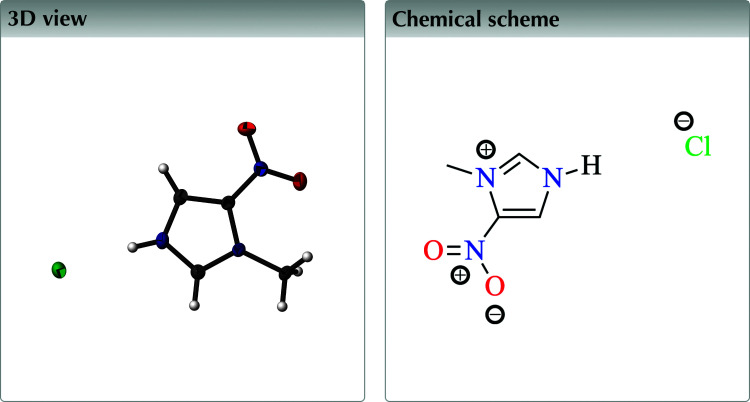

## Structure description

The study of nitro­imidazole-based compounds remains of inter­est due to their appearance on the World Health Organization’s list of essential drugs (Purgato & Barbui, 2012 ▸). Among the numerous functionalized derivatives of imidazoles, 5-nitro­imidazoles have long been known to be effective anti­biotics (Leiros *et al.*, 2004 ▸). Recently, however, 5-nitro­imidazole-based compounds have received renewed attention for the potential treatment of a slew of infectious diseases such as leishmaniasis and tuberculosis (Ang *et al.*, 2017 ▸). A previous report by Bowden & Izadi (1998 ▸) analyzed the anti­bacterial activities of various derivatives of metronidazole, a compound bearing a 5-nitro­imidazole core. In their work, several derivatives of metronidazole were chemically modified and studied with the intent of overcoming some of the disadvantages of 5-nitro­imidazole-based pharmaceuticals (Bowden & Izadi, 1998 ▸). Furthermore, Miyamoto and coworkers reported the synthesis of a new class of nitro­imidazole derivatives to combat drug-resistant strains of infections (Miyamoto *et al.*, 2013 ▸). Hence, with the renewed inter­est in these compounds, fundamental structural analysis of nitro­imidazoles is of importance to the advancement of drug development.

**Figure 1:**
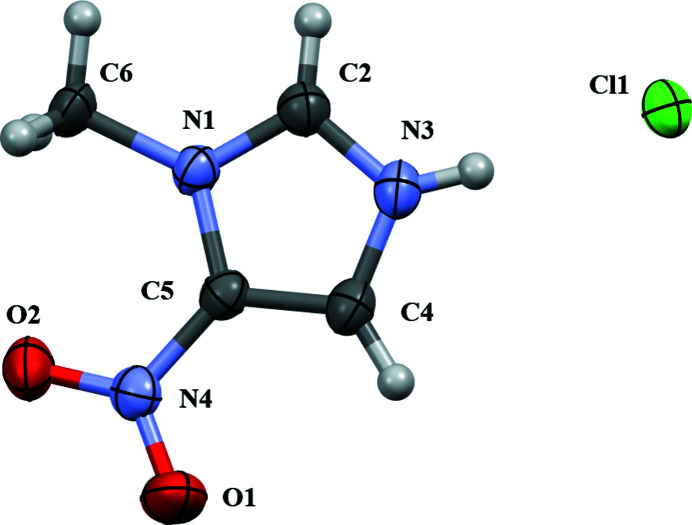
Constituents of the title salt showing the atom-labeling scheme and 50% probability ellipsoids.

Herein we report the crystal structure of 1-methyl-5-nitro­imidazolium chloride (Fig. 1 ▸). While the overall crystalline forces are dominated by the Coulombic inter­actions between ion pairs, non-covalent inter­actions will still play a role in the formation of the crystal (Gavezzotti, 2010 ▸). The amine hydrogen atom, H3, exhibits the shortest hydrogen bond with the chloride anion with a distance of 2.160 (19) Å (Table 1 ▸). The 2-position of imidazolium cations is known to be relatively acidic (Noack *et al.*, 2010 ▸). As such, the central aromatic hydrogen (H2) tends to form shorter inter­actions with anions when compared with the other aromatic H atoms on the heterocyclic cores (Dupont, 2004 ▸). This trend is observed within this structure as well with H2 displaying a shorter inter­action with the anion (2.62 Å) than the other aromatic hydrogen H4 (2.78 Å). As has been observed in related systems, the halide anions surround the cation in distinctive locations facilitating inter­actions with nearly all atoms of the heterocyclic core (Hunt *et al.*, 2006 ▸; Sanchora *et al.*, 2019 ▸; Matthews *et al.*, 2015 ▸). For example, the chloride anion inter­acts with the methyl H atoms (H6*A*, H6*B*, and H6*C*) at distances of 3.14, 2.85, and 2.84 Å, respectively.

**Figure 2:**
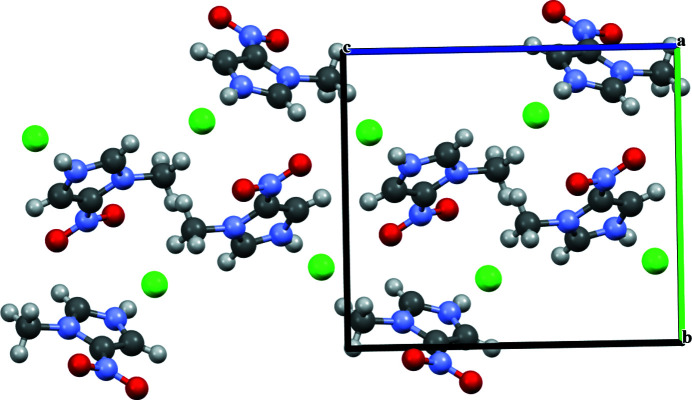
Packing diagram of the title salt.

Nitro moieties are capable of exhibiting a diverse set of non-covalent inter­actions (Bauzá *et al.*, 2019 ▸; Sikorski & Trzybiński, 2013 ▸). Within the title structure, both nitro O atoms (O1 and O2) participate in inter­actions with methyl H atoms H6*A* and H6*B* at distances of 2.56 and 2.90 Å, respectively. No short inter­actions with the aromatic H atoms are observed with the nitro group. The nitro moiety is nearly coplanar to the imidazole ring, with an N4—C5—C4—N3 torsion angle of 6.71 (10)^*o*^. As demonstrated by Bauzá *et al.*, π-holes are present in nitro­aromatics, forming an important set of potential inter­actions (Bauzá *et al.*, 2015 ▸). For the title compound, the chloride anion is inter­acting with both faces of the π-hole of the nitro moiety at distances of 3.33 (10) and 3.37 (10) Å. The packing is shown in Fig. 2 ▸.

## Synthesis and crystallization

The title compound is a hydrolysis product from the synthetic procedure described below, analogous to our previously reported synthesis of 2,3-dimethyl-1*H*-imidazol-3-ium chloride (Anderson *et al.*, 2020 ▸).

In brief, 5-nitro­imidazole and trityl chloride were dissolved in separate 50 ml beakers with toluene. The reactants were then combined in a single-necked 100 ml round-bottom flask equipped with a magnetic stir bar and left to stir for 2 days at room temperature. The solvent was removed under vacuum leaving a white solid residue. This solid was washed twice with tetra­hydro­furan and recovered *via* vacuum filtration. Crystals were grown at room temperature by vapor diffusion with aceto­nitrile as the solvent and tetra­hydro­furan as the anti-solvent. Colorless crystals of the hydrolyzed byproduct reported herein were observed within one week.

## Refinement

For full experimental details including crystal data, data collection and structure refinement details, refer to Table 2 ▸.

The structure emulates a double the volume ortho­rhom­bic *C*-centered cell and is twinned by this symmetry (180° rotation around the real space *a* axis or around the reciprocal direction [ 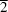 01]). Refinement with the transformation matrix 1 0 0, 0 −1 0, −1 0 −1 yielded a 0.555 (1) to 0.445 (1) twinning ratio.

## Supplementary Material

Crystal structure: contains datablock(s) I. DOI: 10.1107/S2414314622008781/tk4083sup1.cif

Structure factors: contains datablock(s) I. DOI: 10.1107/S2414314622008781/tk4083Isup2.hkl

Supporting information file. DOI: 10.1107/S2414314622008781/tk4083Isup3.cml

CCDC reference: 2204963

Additional supporting information:  crystallographic information; 3D view; checkCIF report

## Figures and Tables

**Table 1:** Hydrogen-bond geometry (Å, °)

*D*—H⋯*A*	*D*—H	H⋯*A*	*D*⋯*A*	*D*—H⋯*A*
N3—H3⋯Cl1	0.856 (19)	2.160 (19)	3.0141 (11)	175.4 (15)
C4—H4⋯Cl1^i^	0.95	2.78	3.6161 (12)	147
C2—H2⋯Cl1^ii^	0.95	2.62	3.4723 (12)	150
C6—H6*B*⋯Cl1^ii^	0.98	2.85	3.7519 (13)	154
C6—H6*C*⋯Cl1^iii^	0.98	2.84	3.6617 (13)	142

Symmetry codes: (i) 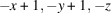 ; (ii) 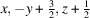 ; (iii) 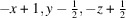 .

**Table 2:** Experimental details

Crystal data	
Chemical formula	C_4_H_6_N_3_O_2_^+^·Cl^−^
*M*_r_	163.57
Crystal system, space group	Monoclinic, *P*2_1_/*c*
Temperature (K)	150
*a*, *b*, *c* (Å)	6.3498 (5), 9.8991 (9), 11.5969 (10)
β (°)	105.817 (3)
*V* (Å^3^)	701.35 (10)
*Z*	4
Radiation type	Mo *K*α
μ (mm^−1^)	0.49
Crystal size (mm)	0.35 × 0.15 × 0.12

Data collection	
Diffractometer	Bruker AXS D8 Quest diffractometer with PhotonII charge-integrating pixel array detector (CPAD)
Absorption correction	Multi-scan (*SADABS*; Krause *et al.*, 2015 ▸)
*T*_min_, *T*_max_	0.659, 0.747
No. of measured, independent and observed [*I* > 2σ(*I*)] reflections	12789, 2681, 2556
*R*_int_	0.037
(sin θ/λ)_max_ (Å^−1^)	0.771

Refinement	
*R*[*F* ^2^ > 2σ(*F* ^2^)], *wR*(*F* ^2^), *S*	0.026, 0.072, 1.08
No. of reflections	2681
No. of parameters	97
H-atom treatment	H atoms treated by a mixture of independent and constrained refinement
Δρ_max_, Δρ_min_ (e Å^−3^)	0.35, −0.24

Computer programs: *APEX3* (Bruker, 2019 ▸), *SAINT* (Bruker, 2019 ▸), *SHELXS97* (Sheldrick, 2008 ▸), *SHELXL2018/3* (Sheldrick, 2015 ▸) *ShelXle* (Hübschle *et al.*, 2011 ▸), *OLEX2* (Dolomanov *et al.*, 2009 ▸), *publCIF* (Westrip, 2010 ▸), and *enCIFer* (Allen *et al.*, 2004 ▸). For the Cambridge Structural Database (CSD), see Groom *et al.* (2016 ▸).

## full crystallographic data

### Crystal data

**Table:**

C_4_H_6_N_3_O_2_^+^·Cl^−^	*F*(000) = 336
*M**_r_* = 163.57	*D*_x_ = 1.549 Mg m^−^^3^
Monoclinic, *P*2_1_/*c*	Mo *K*α radiation, λ = 0.71073 Å
*a* = 6.3498 (5) Å	Cell parameters from 9893 reflections
*b* = 9.8991 (9) Å	θ = 2.8–33.2°
*c* = 11.5969 (10) Å	µ = 0.49 mm^−^^1^
β = 105.817 (3)°	*T* = 150 K
*V* = 701.35 (10) Å^3^	Rod, colourless
*Z* = 4	0.35 × 0.15 × 0.12 mm

### Data collection

**Table:**

Bruker AXS D8 Quest diffractometer with PhotonII charge-integrating pixel array detector (CPAD)	2556 reflections with *I* > 2σ(*I*)
Detector resolution: 7.4074 pixels mm^-1^	*R*_int_ = 0.037
ω and phi scans	θ_max_ = 33.3°, θ_min_ = 2.8°
Absorption correction: multi-scan (SADABS; Krause *et al.*, 2015)	*h* = −8→9
*T*_min_ = 0.659, *T*_max_ = 0.747	*k* = −15→14
12789 measured reflections	*l* = −16→17
2681 independent reflections	

### Refinement

**Table:**

Refinement on *F*^2^	Primary atom site location: structure-invariant direct methods
Least-squares matrix: full	Secondary atom site location: difference Fourier map
*R*[*F*^2^ > 2σ(*F*^2^)] = 0.026	Hydrogen site location: mixed
*wR*(*F*^2^) = 0.072	H atoms treated by a mixture of independent and constrained refinement
*S* = 1.08	*w* = 1/[σ^2^(*F*_o_^2^) + (0.039*P*)^2^ + 0.1001*P*] where *P* = (*F*_o_^2^ + 2*F*_c_^2^)/3
2681 reflections	(Δ/σ)_max_ = 0.001
97 parameters	Δρ_max_ = 0.35 e Å^−^^3^
0 restraints	Δρ_min_ = −0.24 e Å^−^^3^

### Special details

**Table:**

Geometry. All esds (except the esd in the dihedral angle between two l.s. planes) are estimated using the full covariance matrix. The cell esds are taken into account individually in the estimation of esds in distances, angles and torsion angles; correlations between esds in cell parameters are only used when they are defined by crystal symmetry. An approximate (isotropic) treatment of cell esds is used for estimating esds involving l.s. planes.
Refinement. Refined as a two-component twin. H atoms attached to carbon atoms were positioned geometrically and constrained to ride on their parent atoms. C—H bond distances were constrained to 0.95 Å for aromatic C—H moieties with *U*_iso_(H) = 1.2 × *U*_eq_(C), and to 0.98 Å for CH_3_ moieties with *U*_iso_(H) = 1.5 × *U*_eq_(C). The N—H proton on N3 was located as residual electron density and allowed to refine freely.

### Fractional atomic coordinates and isotropic or equivalent isotropic displacement parameters (Å^2^)

**Table:**

	*x*	*y*	*z*	*U*_iso_*/*U*_eq_	
Cl1	0.75167 (5)	0.72586 (3)	0.07499 (2)	0.02027 (7)	
O1	−0.1250 (2)	0.37864 (11)	0.13424 (9)	0.0313 (2)	
O2	−0.14844 (16)	0.45993 (11)	0.30478 (8)	0.02609 (19)	
N3	0.42357 (17)	0.61303 (11)	0.19454 (9)	0.02019 (19)	
H3	0.512 (3)	0.6439 (18)	0.1567 (14)	0.019 (4)*	
N1	0.25774 (17)	0.58619 (10)	0.33532 (8)	0.01734 (17)	
N4	−0.05494 (18)	0.44564 (10)	0.22517 (8)	0.01998 (18)	
C5	0.14730 (19)	0.51334 (11)	0.23575 (9)	0.01723 (18)	
C4	0.2514 (2)	0.53027 (13)	0.14779 (10)	0.0202 (2)	
H4	0.211185	0.491815	0.069785	0.024*	
C2	0.4252 (2)	0.64545 (12)	0.30717 (10)	0.0202 (2)	
H2	0.530167	0.702184	0.358999	0.024*	
C6	0.2176 (2)	0.59302 (13)	0.45524 (10)	0.0224 (2)	
H6A	0.081178	0.642222	0.449455	0.034*	
H6B	0.339456	0.640052	0.510943	0.034*	
H6C	0.205596	0.501290	0.484604	0.034*	

### Atomic displacement parameters (Å^2^)

**Table:**

	*U*^11^	*U*^22^	*U*^33^	*U*^12^	*U*^13^	*U*^23^
Cl1	0.02085 (12)	0.01942 (11)	0.02158 (11)	−0.00029 (9)	0.00752 (10)	0.00269 (9)
O1	0.0386 (6)	0.0293 (5)	0.0243 (4)	−0.0130 (4)	0.0060 (4)	−0.0054 (3)
O2	0.0224 (4)	0.0351 (5)	0.0225 (4)	−0.0009 (4)	0.0090 (3)	0.0033 (3)
N3	0.0207 (4)	0.0232 (5)	0.0186 (4)	0.0012 (4)	0.0086 (4)	0.0026 (3)
N1	0.0206 (4)	0.0166 (4)	0.0161 (4)	−0.0004 (3)	0.0071 (3)	−0.0011 (3)
N4	0.0217 (4)	0.0195 (4)	0.0182 (4)	−0.0011 (4)	0.0045 (3)	0.0033 (3)
C5	0.0194 (5)	0.0169 (4)	0.0152 (4)	0.0010 (4)	0.0044 (4)	0.0010 (3)
C4	0.0223 (5)	0.0229 (5)	0.0160 (4)	0.0016 (4)	0.0060 (4)	0.0014 (4)
C2	0.0214 (5)	0.0200 (5)	0.0199 (5)	−0.0011 (4)	0.0068 (4)	−0.0001 (4)
C6	0.0306 (6)	0.0232 (5)	0.0162 (4)	−0.0047 (4)	0.0110 (4)	−0.0038 (4)

### Geometric parameters (Å, º)

**Table:**

O1—N4	1.2223 (14)	N4—C5	1.4239 (16)
O2—N4	1.2343 (13)	C5—C4	1.3687 (16)
N3—H3	0.856 (19)	C4—H4	0.9500
N3—C4	1.3553 (16)	C2—H2	0.9500
N3—C2	1.3423 (15)	C6—H6A	0.9800
N1—C5	1.3797 (14)	C6—H6B	0.9800
N1—C2	1.3307 (16)	C6—H6C	0.9800
N1—C6	1.4822 (14)		
			
C4—N3—H3	125.4 (11)	N3—C4—C5	106.06 (10)
C2—N3—H3	125.5 (11)	N3—C4—H4	127.0
C2—N3—C4	108.98 (10)	C5—C4—H4	127.0
C5—N1—C6	129.12 (10)	N3—C2—H2	125.1
C2—N1—C5	106.44 (10)	N1—C2—N3	109.79 (11)
C2—N1—C6	124.29 (10)	N1—C2—H2	125.1
O1—N4—O2	124.85 (12)	N1—C6—H6A	109.5
O1—N4—C5	115.92 (10)	N1—C6—H6B	109.5
O2—N4—C5	119.21 (10)	N1—C6—H6C	109.5
N1—C5—N4	123.97 (10)	H6A—C6—H6B	109.5
C4—C5—N1	108.73 (10)	H6A—C6—H6C	109.5
C4—C5—N4	126.94 (10)	H6B—C6—H6C	109.5
			
O1—N4—C5—N1	−175.66 (11)	C4—N3—C2—N1	−0.17 (14)
O1—N4—C5—C4	11.97 (18)	C2—N3—C4—C5	0.12 (14)
O2—N4—C5—N1	5.77 (16)	C2—N1—C5—N4	−173.64 (11)
O2—N4—C5—C4	−166.59 (12)	C2—N1—C5—C4	−0.08 (13)
N1—C5—C4—N3	−0.02 (13)	C6—N1—C5—N4	10.81 (18)
N4—C5—C4—N3	173.29 (11)	C6—N1—C5—C4	−175.63 (11)
C5—N1—C2—N3	0.15 (13)	C6—N1—C2—N3	175.98 (10)

### Hydrogen-bond geometry (Å, º)

**Table:**

*D*—H···*A*	*D*—H	H···*A*	*D*···*A*	*D*—H···*A*
N3—H3···Cl1	0.856 (19)	2.160 (19)	3.0141 (11)	175.4 (15)
C4—H4···Cl1^i^	0.95	2.78	3.6161 (12)	147
C2—H2···Cl1^ii^	0.95	2.62	3.4723 (12)	150
C6—H6*B*···Cl1^ii^	0.98	2.85	3.7519 (13)	154
C6—H6*C*···Cl1^iii^	0.98	2.84	3.6617 (13)	142

Symmetry codes: (i) −*x*+1, −*y*+1, −*z*; (ii) *x*, −*y*+3/2, *z*+1/2; (iii) −*x*+1, *y*−1/2, −*z*+1/2.
